# The Duty of the Doctor to the Dying

**Published:** 1877-12-20

**Authors:** 


SOUTHERN MEDICAL RECORD.

VOL. VII. ATLANTA, GA., DECEMBER 20, 1877. NO. 12.

THOMAS S. POWELL, M.D.,

W. T. GOLDSMITH, M. D.,

R. C. WORD, M. D.,

} EDITORS.

R. C. WORD, Business Manager.

SUBSCRIPTION: TWO DOLLARS PER ANNUM, IN ADVANCE.

☞ All communications, and letters on business connected with THE RECORD for 1877 must be addressed to the Business Manager.

### Original and Selected Articles.

### THE DUTY OF THE DOCTOR TO THE DYING.

BY A VIRGINIA PRACTITIONER.

A recent death-bed scene has awakened inquiries in my mind as to the duties and responsibilities of physicians to the dying, which have impressed me with a sense of obligation never before so profoundly appreciated. A brief allusion to the circumstances which aroused these reflections, may not be amiss.

Mrs. ——, a highly respectable and intelligent lady, the wife of a fond husband, and the mother of four young and lovely children, was sinking under the wasting effects of pulmonary consumption. A judicious course of treatment had arrested the progress of the disease, and for twelve months given bright hopes of entire recovery. The hectic had ceased—tubercles no longer were discharged in the expectoration —flesh and vigor had returned, and she presided with efficiency over her household affairs. The cares of an infant, seven or eight months old—sudden domestic trouble—a violent catarrh, all came upon her in the first relaxing hours of summer, and rapid degeneration of tuberculous masses ensued, and she withered almost as fast as the new-mown hay. The final scene rapidly approached, and the night before its close, the rapid degeneration of and discharge of tuberculous abscesses so obstructed the bronchia that for an hour or more every respiration seemed as though it were the last she could make. Ammonia, alcohol, change of position, exciting inhalations sustained her until the abscess was emptied, and she had a respite.

After a few moments of repose, her mind seemed to seize upon the startling premonitions of her situation, and she realized, for the first time, that death was marching with stately steps into the citadel of life, and would soon reign supremely over all her vital powers, unless his progress could speedily be stayed by some giant hand. Then transpired a scene which beggars description. The most earnest and searching questions as to her true condition were propounded to her physician. No evasion or prevarication would be brooked for an instant. Am I dying? How long can I live? Shall I be required to pass through such an agony again before I go? Did my husband and dear little ones see my struggles? Will you pray God to give me grace to meet the next onset of the monster? Let me give my husband my parting counsel, and final adieu. Bring my children, that I may embrace and kiss them a final farewell. Tell me, Doctor, of the promises and consolations of the Bible. Let me have something to avert the awful sufferings and agonies through which I have passed. Tell me plainly, must I die to-night? etc., etc.

These incidents occupied several hours. Falling into a sweet slumber, she rested for an hour or more, to be aroused by premonitions of another paroxysm of suffocation. She took in the situation at once, and manifested unusual agitation and alarm.

"Oh, Doctor! spare me such a trial. I am not afraid to die. I know in whom I have believed; but to suffocate! Oh! can't you give me something to prevent it?"

The doctor was using every means in his power to meet the exigencies of the case, but all in vain. As though an abscess had ruptured in every bronchial tube, both lungs were soon filled with viscid and tenacious mucous and pus, and the rushing and cracking sound of the air, as it broke through this obstruction, could be heard far out in the yard. Change of position, inhalations, frictions, etc., etc., availed nothing, and nothing could be swallowed. This terrible, awful struggle lasted for an hour, when, by a mighty effort, she exclaimed:

"Oh, Doctor! cut my throat and let me die."

When assured that all was being done that could be, despair brooded over her features, and her eyes became fixed; her face livid; her body wet with cold sweat, and it was hoped the end was at hand. But another hour of death-struggle passed, when again she roused up, and begged that "something should be given to stay her torture or end her life, for she was not afraid to die." By a spasmodic effort, she imbibed a little ammonia and brandy, fell upon her pillow, and breathed no more.

To one who has himself experienced the consolations of religion *in extremis*—who has often rehearsed the promises and precepts of the gospel to the dying Christian—it were a work of supererrogation to point out his duty in such an emergency as the above, when his duties are confined alone to the spiritual aspect of the case. But whether Christian or infidel, what to do to meet the emergencies of the physical condition, is the great problem for solution.

There is a case in which recovery, or even temporary relief, cannot reasonably be hoped for. Death is on his swift march to a final possession of the citadel of life, and the vital powers are making a most desperate and agonizing struggle to maintain their hold. The suffering is of such extreme character as to make excrutiating pain a most welcome exchange. The person has made preparation for her exit from time, and the rendition of her final account. She has disposed of her worldly effects, given her parting counsel to her children, bidden adieu to her friends, and was willing and anxious to depart. Her struggles are such that friends and attendants close their eyes, or avert their faces, while one who had witnessed many a death-bed scene fainted while she strove in vain to afford relief. The struggle goes on, despite all efforts to arrest it, and it culminates in such torturing agony as to extort the cry, "Oh, Doctor, cut my throat and let me die!"

What should be done? Nothing could be swallowed. Camphor, ammonia, alcohol, cologne, etc., were given by inhalation; doors and windows were all opened; the room cleared of needless attendants; the air agitated with fans; stimulating frictions used; position frequently changed, and all to no purpose. Would it be proper to administer one or two inspirations of chloroform, under the circumstances? One or two inspirations would assuredly have put an end to the struggle, and closed the painful scene. But would it have been legitimate? Nothing short of some agency which would have overpowered the vital energies and produced a state of unconsciousness, would have met the requirements, and to have done this would have arrested the struggle as well as the life-tide.

Under the circumstances, the attending physician did nothing to accomplish these ends, and the result was that the terrible torture was prolonged for hours. Should the same course be pursued under like circumstances? The more the writer contemplates that scene, the more bewildered he becomes, as he attempts to decide what he would do should he be subjected to a similar trial. How could he resist the appeal for relief, though relief and death are synonymous terms? Would he not reproach himself if the former were secured at the sacrifice of only *an hour's living death?* Were he to act under the golden rule, to "Do unto others as you would that others should do unto you," he would not hesitate to stop the agonizing torture, even at the expense of the few remaining hours of life. But would even this compensate the mental conflict which a conviction of certainly shortening one's existence would produce? How often is it the case, that the surgeon, convinced that a tumor unremoved must certainly result in death within a year or two, and that an operation may be successful or immediately fatal, proceeds, at the instance of the patient, to operate, and is arrested in his work by the advent of death? When it is evident a patient must die in a few hours, and she is suffering tortures more agonizing than death, why may not the doctor administer relief, though death is the inevitable result? I say *inevitable*, because it would so be. in all human probability. I am sure this is a question demanding a serious consideration, which it is hoped your contributors may give it.

